# Pipe Cleaners as a Low-Cost and Versatile Educational Tool for Teaching Vascular Anatomy

**DOI:** 10.1007/s40670-021-01224-y

**Published:** 2021-02-02

**Authors:** Thomas Franchi

**Affiliations:** 1 The Medical School, The University of Sheffield, Beech Hill Road, Sheffield, S10 2RX UK; 2 Department of Biomedical Science, The University of Sheffield, Firth Court, Sheffield, S10 2TN UK

**Keywords:** Pipe cleaners, Vascular anatomy, Anatomical education, Educational technology, Teaching methods, Cost-effective education

## Abstract

Teaching and learning vascular anatomy can be challenging for both educators and students. Cadaveric vasculature is difficult to visualise whilst models are costly and fixed in position. This paper proposes the use of pipe cleaners as a low-cost and versatile educational tool for use by both anatomy educators and students.

The process of both teaching and learning anatomy has been subject to scrutiny for some time, with innovative teaching methods and the incorporation of novel technologies being at the forefront of debate regarding new approaches [1]. Ongoing limitations surrounding anatomical education, such as decreasing exposure to cadavers, have led educators to employ a broader range of pedagogies in the pursuit of anatomical simplification and effective learning [2].

This article presents a novel educational tool for teaching vascular anatomy, which is simultaneously low cost and versatile, and can be utilised in any setting. Teaching and learning vascular anatomy, particularly in areas of the human body with vast branching networks or where common variations can be found, can be challenging for both educators and students. The beauty of anatomical models is that they can transform a complicated structure into a simpler, more visually welcoming form. Indeed, the use of models not only allows for a clearer view of a structure, away from the complexity-based and ethical-based issues associated with cadavers, but also gives students in all institutions the opportunity to study anatomy in a three-dimensional, physical form, irrespective of exposure to dissection [3].

In view of this, a novel teaching method resource was designed, prototyped and trialled in teaching sessions. A model of the abdominal aorta, with its three anterior visceral branches, was produced using kitchen roll and pipe cleaners (Fig. 1). Alongside this, individual models of each main branch, such as the superior mesenteric artery (Fig. 2), were made using toilet roll and pipe cleaners. Each vascular division was indicated by a differently coloured pipe cleaner, connected by simple twisting. A further level of information was provided by individual tags attached to each pipe cleaner, which indicate the specific vessel name. Oral feedback was collected from second-year undergraduate biomedical science students.

**Fig. 1:**
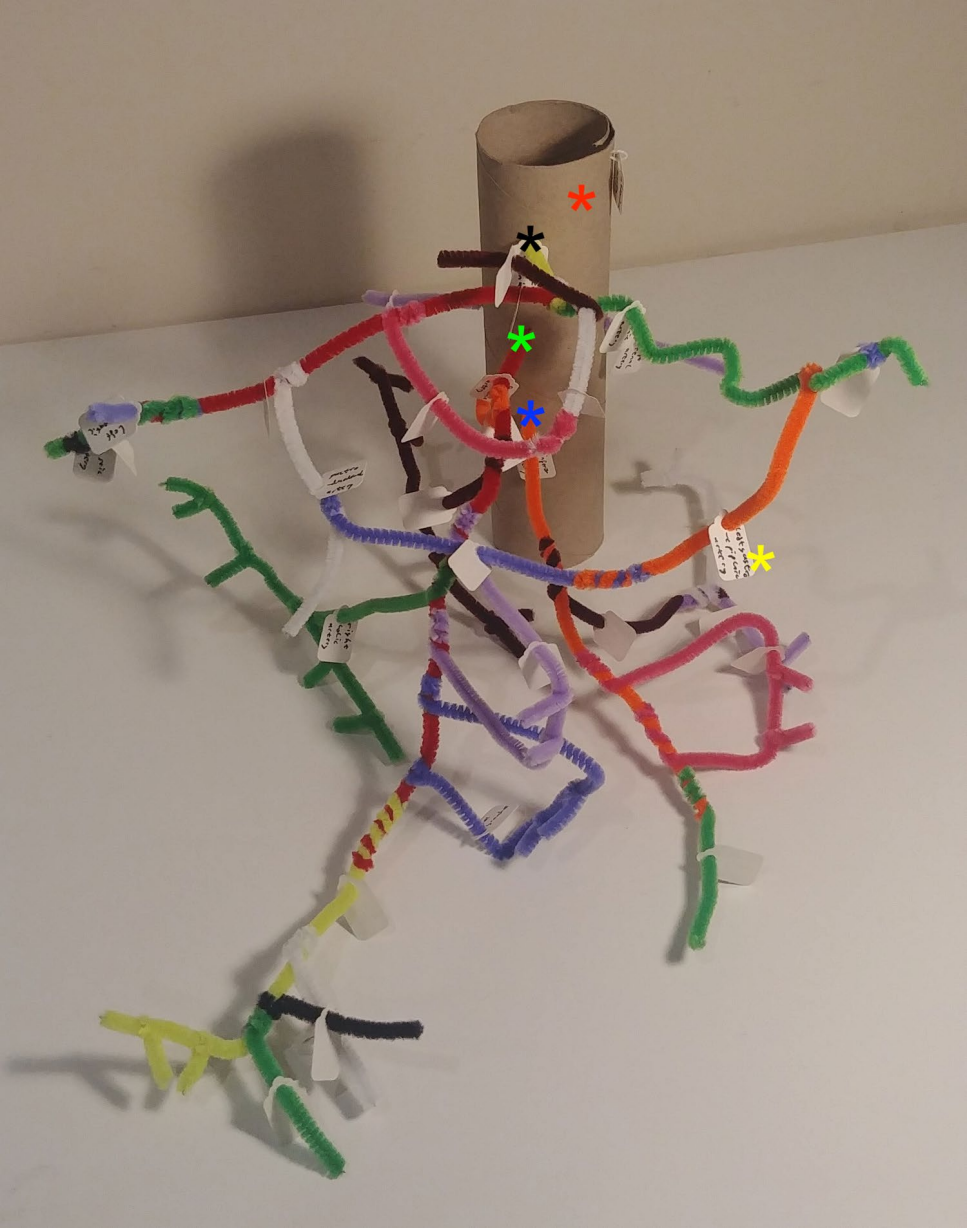
Model of the abdominal aorta (toilet roll) with its three anterior visceral branches (pipe cleaners), which further divide. Red * = abdominal aorta, black * = origin of coeliac trunk, green * = origin of superior mesenteric artery, blue * = origin of inferior mesenteric artery, yellow * = tag to indicate vessel name

**Fig. 2:**
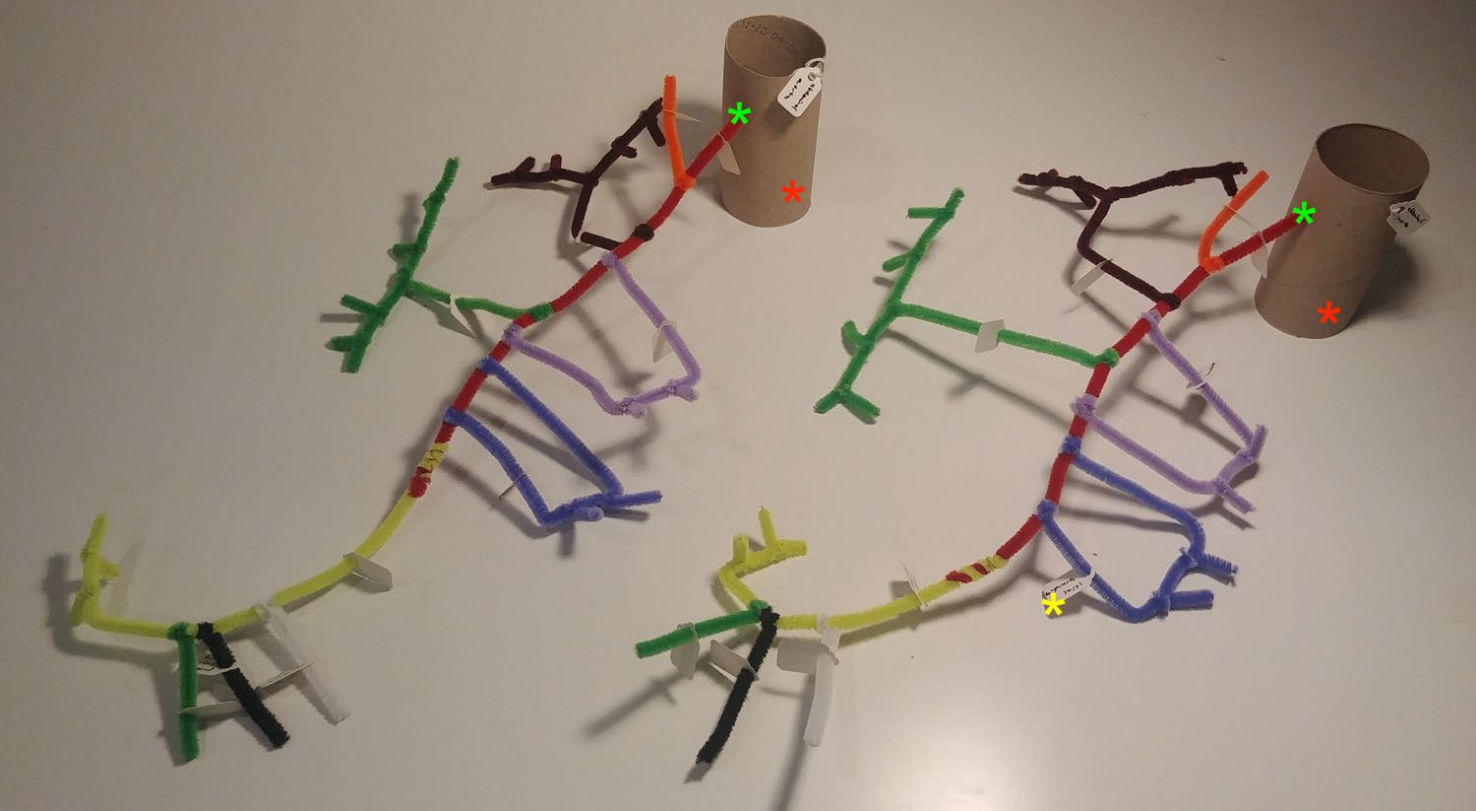
Models of the superior mesenteric artery, branching off the abdominal aorta (toilet toll), with its branches (pipe cleaners). Red * = abdominal aorta, green * = origin of superior mesenteric artery, yellow * = tag to indicate vessel name

There are numerous benefits to this method, which make it a different and worthwhile technique. When students first saw these colourful models made of pipe cleaners, many reacted with interest and a sense of excitement. Simply using an unusual medium in itself engaged students, before ‘teaching’ even began. The oral feedback received in conversation with students was highly positive, with students commonly citing kinaesthetic learning aspects which this method enabled. An ability to hold, move and manipulate the models proved beneficial to students’ understanding and spatial awareness:

Being able to hold and change the shape of the model really helped me to understand the 3D structure and course of the vessels. – Student A.

It was clear from the teaching sessions that students were more engaged with these models than with typical anatomic models, which are fixed in position and offer only limited scope for interaction and manipulation. The malleable nature of this tool lends itself to be added to skeletons, for example, to demonstrate their position or anatomical course. The individually coloured pipe cleaners can aid students with visual learning styles and can further be used to demonstrate common variations within one model, where each variation is represented by a separate colour. These models can be used in any context given their cheapness, ease of use, reusability and ability to personalise, such as dissection adjuncts, tutorial props, seminar aids, virtual lecture demonstrations or assignment tools. Finally, the low-cost and readily available materials used to produce these models means that students can be encouraged to make them at home. This active process can allow students to learn whilst building the models and can further be used for revision purposes, allowing ongoing learning beyond the classroom:

I struggle to learn the branches of blood vessels when I revise with textbooks. Having a model that I can make at home from cheap pipe cleaners will change the way I study anatomy. – Student B.

Similar methods can of course also be used in the teaching and learning of other areas of vascular anatomy, as well as in the study of the divisions of nerves or even muscle origins and insertions. Indeed, previous authors have highlighted the use of pipe cleaners in teaching the brachial plexus, with high levels of student satisfaction [4]. Reasonable limitations of this method include the generally inaccurate representation of structures in relation to their size, shape and texture. However, given the purpose of this pedagogical adjunct, these are not of major significance. It is hoped that this educational tool will be adopted by both educators and students and that it will prove beneficial in furthering anatomical understanding and knowledge.
